# Identification of squalene epoxidase in triterpenes biosynthesis in *Poria cocos* by molecular docking and CRISPR-Cas9 gene editing

**DOI:** 10.1186/s12934-024-02306-3

**Published:** 2024-01-25

**Authors:** Xiao-liu Liu, Jing Xie, Zhen-ni Xie, Can Zhong, Hao Liu, Shui-han Zhang, Jian Jin

**Affiliations:** 1 Institute of Chinese Medicine Resources, Hunan Academy of Chinese Medicine, Changsha, 410013 China; 2 Hunan Academy of Chinese Medicine, Hunan University of Traditional Chinese Medicine, Changsha, 410208 China

**Keywords:** *Poria cocos*, Squalene epoxidase, CRISPR/Cas9 system, Triterpenoids, Gene function

## Abstract

**Background:**

Squalene epoxidase is one of the rate-limiting enzymes in the biosynthetic pathway of membrane sterols and triterpenoids. The enzyme catalyzes the formation of oxidized squalene, which is a common precursor of sterols and triterpenoids.

**Result:**

In this study, the squalene epoxidase gene (*PcSE*) was evaluated in *Poria cocos*. Molecular docking between *PcSE* and squalene was performed and the active amino acids were identified. The sgRNA were designed based on the active site residues. The effect on triterpene synthesis in *P. cocos* was consistent with the results from ultra-high-performance liquid chromatography-quadruplex time-of-flight-double mass spectrometry (UHPLC-QTOF-MS/MS) analysis. The results showed that deletion of *PcSE* inhibited triterpene synthesis. In vivo verification of *PcSE* function was performed using a PEG-mediated protoplast transformation approach.

**Conclusion:**

The findings from this study provide a foundation for further studies on heterologous biosynthesis of *P. cocos* secondary metabolites.

**Supplementary Information:**

The online version contains supplementary material available at 10.1186/s12934-024-02306-3.

## Highlights

- The gene of squalene epoxidase in* P. cocos* was identified.
- Molecular docking was used to predict the amino acid active site of *PcSE*.
- Establishment of a CRISPR/Cas9 gene editing system in* P. cocos*.
- Function validation of *PcSE* on triterpene synthesis by gene deletion.

**Supplementary Information:**

The online version contains supplementary material available at 10.1186/s12934-024-02306-3.

## Introduction

*Poria cocos* (Schw.) Wolf is a parasitic fungus found on the roots of pine trees. It belongs to the Poriferae family of fungi, and its dried nucleus is known as Fu Ling in China [28]. *P. cocos* is edible and has high medicinal value. It was initially reported as an ancient Chinese medical ingredient, ‘Shen Nong Ben Cao Jing’ [1]. The dry sclerotium of *P. cocos* has been used for more than 2000 years in traditional Chinese medicine [35]. *P. cocos* has been widely explored by chemists and biologists because of its role in production of several bioactive components and its potential as a nutritional supplement or for the preparation of pharmaceutical components [2]. Two main secondary metabolites, triterpenes and polysaccharides, have been isolated from *P. cocos* to date.

The steroidal triterpenoids present in *P. cocos* are mainly classified into four categories: steroid-8-ene-type triterpene acid, steroid-7,9(11)-diene-type triterpene acid, 3,4-open-loop-woolly steroid-7,9(11)-diene-type triterpene acid, and 3,4-open-loop-woolly steroid-8-ene-type triterpene acid [27, 36, 37] These compound have several effects, such as prevention of renal tubular fibrosis [26], inhibition of tyrosinase [11], anti-inflammatory activity [15], anti-tumor activity [29], and prevention of Alzheimer's disease [6]. Notably, these compounds are key active ingredients in *P. cocos*. However, the levels of triterpenes are low in *P. cocos* medicinal ingredients [39]. In addition, the triterpenes are isomeric or structurally similar and difficult to isolate and purify. The low content and rareness of *P. cocos* triterpene compounds, currently costing approximately 100,000 Yuan/g, markedly limits the development of new drugs based on *P. cocos* triterpenes.

Squalene epoxidase catalyzes the oxidation of squalene to generate 2–3-epoxysqualene in the *P. cocos* triterpene synthesis pathway. The enzyme is a key rate-limiting enzyme in the synthesis of triterpenoids and is an important component in understanding the synthetic biology of triterpenoids [16, 17]. In addition, *SE* is a key enzyme in the synthesis of ergosterol, a constituent of cell membranes, and the absence or silencing of *SE* inhibits ergosterol synthesis [20]. Cloning and expression analysis of *SE* genes have been carried out in the commonly used traditional Chinese medicine resources such as *Panax schinseng* [34], *Panax notoginseng* [32] and *Gynostemma pentaphyllum* [8]. However, studies on the role of *SE* gene in *P. cocos* have not been reported. In our previous study, we evaluated the triterpene profile of *P. cocos* by LC–MS [12, 13]. Moreover, the triterpene synthesis pathway in *P. cocos* was evaluated through genomic data annotation and the sequence of *PcSE* gene was cloned from the cDNA of *P. cocos*. Genetic evolution analysis and structural domain prediction were carried out to explore the biotransformation. The findings showed that positions 155–419 comprised a SE cyclooxygenase structural domain, which was tentatively predicted to play the role of squalene cyclooxygenase enzyme. However, the function of *PcSE* gene should be explored further through experimental studies.

CRISPR/Cas9 technology is a novel gene-targeted editing technology. This technology uses a segment of specific sgRNA to guide the nucleic acid endonuclease Cas to a target gene, thus accomplishing DNA identification and editing in a low-cost and targeted manner [22]. As a result, it is widely used for gene function verification. It is currently used for gene editing in various organisms, including fungi, but this technology has not been applied in study of *P. cocos*. Lack of efficient genetic transformation techniques limits research on regulation of active ingredient synthesis and restricts studies on improving *P. cocos* growth and development of effective molecular breeding strategies [31].

In this study, *PcSE* was cloned based on *P. cocos* genomic data. A molecular docking approach was used to predict the active site amino acids of the protein. These active site residues were used to design the sgRNA sequence and the gene editing system was optimized. Gene editing was performed with *PcSE* as the target gene, and performed in vivo functional validation. Genes that encode key enzymes were identified and *P. cocos* triterpene synthesis pathway was explored.

## Material and methods

### Strains and culture media

*P. cocos* strain Xiangjing 28 was used for CRISPR/Cas9 gene editing experiments. The fungus strain was collected from Jingzhou County, Hunan Province, China (N 26^◦^43′36.38′′, E 109^◦^41′15.34′′), and firstly, a soft-bristle brush was used to remove the residual soil on the surface of the kernel, excise the yellowish-brown epidermis, and spray it with 75% alcohol; then, a sterile surgical blade was used to excise the sprayed alcohol part on the ultra-clean workbench, and tweezers were used to clip the white kernel on solid fungal medium, and then the kernel was cultured in RXZ-380 Intelligent Artificial Climate Chamber (Ningbo Jiangnan Instrument Factory) under 28 °C. The strain was preserved in Institute of Chinese Medicine Resources, Hunan Academy of Chinese Medicine. It was preserved in a fungal medium (ZJ; g/L, 15 glucose, 1 KH_2_PO_4_, 0.5 MgSO_4_·7H_2_O, 2 yeast extract, 5 peptone and 20 agar). *Escherichia coli* strain DH5*α* (Tiangen Biotech Co., Ltd. (Beijing, China)) was used for construction of recombinant plasmids and stored at -80 °C. *E. coli* strains were cultured on Luria–Bertani (LB) medium supplemented with Ampicillin (50 μg mL^−1^) as a selection marker at 37 °C for vector propagation. *P. cocos* strain was grown in ZJ medium at 28 °C as described in a previous study [3].

### Chemicals and reagents

16α-hydroxydehydrotrametenolic acid (HDTRA), poricoic acid B (PAB, CAS No. 137551-39-4), dehydrotumulosic acid (DTUA, CAS No. 6754-16-1), polyporenic acid C (PAC, CAS No. 465-18-9), dehydropachymic acid (DPA, CAS No. 77012-31-8), poricoic acid AM (PAAM, CAS No. 151200-92-9), 3-O-acetyl-16α-hydroxydehydrotrametenolic acid (AHDTRA, CAS No. 168293-14-9), 3-O-acetyl-16α-hydroxytrametenolic acid (AHTRA, CAS No. 168293-13-8) and pachymic acid (PA, CAS No. 29070-92-6) were purchased from Chroma Biotechnology Co., Ltd (Chengdu, China). All other reagents were of analytical grade.

### RNA isolation, cDNA synthesis, gene cloning and bioinformatics analysis

*P. cocos* strains preserved in the incubator were punched using a 1 mL lance tip, inoculated on fresh ZJ medium plates, and the mycelium was incubated at 28 °C. Fresh *P. cocos* mycelium cultured for 7 days was collected and picked up in a mortar with forceps, quickly poured into liquid nitrogen for grinding, and placed in a 2 mL EP tube for use before the powder melted. Total RNA of *P. cocos* mycelia was extracted using RNAprep Pure Plant Plus Kit Polysaccharide Polyphenol Plant Total RNA Extraction Kit (Tsingke Biotech Co., Ltd. (Beijing, China)) according to the instructions (room temperature). RNA concentration and purity were detected using a ScanDrop 100 (Thermo Fisher Scientific Co., Ltd, Waltham, MA, USA), RNA band integrity was detected by 1% agarose gel electrophoresis, and RNA samples were placed at − 80 ℃ for backup. RNA reverse transcription was performed to synthesize the first strand cDNA using 1 μL of *P. cocos* total RNA as a template according to the GoldenstarTM II RT6 cDNA Synthesis Kit Reverse Transcription Kit instructions (Tsingke Biotech Co., Ltd. (Beijing, China)). and gene cloning of *P. cocos* mycelia were based on the whole genome sequencing data of *P. cocos*, the genome database of *P. cocos* was analyzed to obtain the complete open reading frame (ORF) of the *PcSE* gene, and the full-length primers for *PcSE* were designed online using NCBI software, and the specific cDNA reverse transcription upstream primers (*PcSE*-F, ATGTCGCCCTACGACGTGCTCAT) and downstream primer (*PcSE*-R, TCACCACCATCGGATCTCCGTCC. PCR was performed using Gold Medal Mix (green), and the PCR product was purified by the DNA Gel Recovery Kit TSP601 (Tsingke Biotech Co., Ltd. (Beijing, China)), and ligated to the T vector at 25 ℃ for 5 min, and then ligated products were transformed into *E. coli* Trans1-T1 receptor cells and coated on LB plates containing ampicillin (Ampicillin, 100 mg/L) and incubated at 37 ℃ for 12–16 h. Positive clones were selected by colony PCR and sent to Tsingke Biotech Co., Ltd. for sequencing, and then preserved to determine that there was no mutation in the strains.

Sequence matching analysis was performed using DNAman Version 6 (Lynnon Biosoft, San Ramon, CA, USA). Open reading frames (ORFs) in the sequences were searched using NCBI ORF Finder (https://www.ncbi.nlm.nih.gov/orffinder/). Single protein structural domain prediction was performed using the CDD tool from the NCBI website (https://www.ncbi.nlm.nih.gov/Structure/cdd/wrpsb.cgi). Three-dimensional (3D) protein structures were modeled using AlphaFold tool (https://www.alphafold.ebi.ac.uk/), the reliability of protein structure was evaluated by Ramachandran plot and VERFY-3D scores. Protein pre-processing and search for potential binding sites in the protein were performed using Discovery studio 2019 software. Define the active site as center_x: 3.840; center_y: − 5.216; center_Z: − 3.108 with box sizes size_x:33; size_y:33; size_z:33 and subsequently export the docking box file config.txt. Small molecule ligands were prepared using ChemDraw 14.0 software. Chamdraw 14.0 software was used to draw the 2D structure and Chamdraw3D was used to convert the 2D structure into a 3D structure and save it as mol2. The 3D structure was imported and the small molecules were processed in the Prepare Ligands module of Molecules, mainly by minimizing the energy of the small molecules and giving them the CHARMm. The prepared small molecules were obtained and saved in mol2 format. Molecular docking of the prepared ligand compound structure to the macromolecular receptor was performed using Autodock-vina. Molecular docking between *PcSE* and squalene was performed using Autodock-vina 1.1.2 software. PyMOL 2.3.0 was utilized to visualize the processed proteins and docking results [10, 30].

### Construction of Cas9 and sgRNA knockout plasmids

The pFC332 plasmid [19] was used as the original CRISPR/Cas9 backbone for genome editing of *P. cocos*. *PcSE* knockout vector pFC332-U6-sgRNA_*PcSE*_-Cas9 was generated by double digestion of plasmid pFC332 at *BglII* and *PacI* sites with insertion of *P. cocos* U6 promoter, gRNA scaffold, terminator and specific sgRNA-targeted knockout sites. The hygromycin was used as a selection marker. We optimized the endogenous U6 promoter of *P. cocos* and performed promoter analysis using Gene promoter Miner (http://gpminer.mbc.nctu.edu.tw/index.php) to ensure high specificity and reduce low off-target efficiency. Subsequently, the two specific sgRNA targeting knockout sites containing 20 bp bases were inserted using DeepHF (http://www.deephf.com/index/#/Predict) for sgRNA design [5]. The knockout vector pFC332-U6-sgRNA_*PcSE*_-Cas9 was then transformed to *E. coli* Top 10 for propagation.

### PEG-mediated protoplast transformation

The PEG-mediated protoplast transformation was performed as previously described with minor modifications [21, 33]. Protoplasts were digested with 1.5% lysozyme (Guangdong Culture Collection Center, Guangzhou, China) at 80 rpm for 2 h at 28 °C and filtered through repeated washing with 0.6 M mannitol. Protoplasts were resuspended by washing with MTC buffer (0.6 M mannitol, 50 mM CaCl_2_, 10 mM Tris Cl pH 7.5) at a density of 10^7^ protoplasts per mL. Each 100 µL of protoplasts was mixed with 5–10 µg of pFC332-U6-sgRNA_*PcSE*_-Cas9 knockout vector plasmid and incubated in an ice bath for 20 min. A volume of 600 µL of PTC buffer (PEG 4000 45%, 50 mM CaCl_2_, 10 mM Tris–Cl pH 7.5) was slowly added to the protoplasts and the mixture was immediately incubated on ice for 20 min, followed by addition of 200 µL of pre-cooled MTC buffer under thorough mixing. The mixture was transferred to ZJ solid medium containing 0.6 M mannitol, ergosterol (80 μg mL^−1^) and Hygromycin B (50 μg mL^−1^) and incubated until the mycelium grew out of the medium to identify resistance markers for detection of transformants with target mutations. Vector plasmid transfer verification was performed using plasmid-specific primers, namely U6-F (TATACTGGGCCCGGGAAGAT) and U6-R (AACTTGGGCGGTGATTCTGC). Gene sequence knockout primers *PcSE*-F1 (CCCCAAGCCACTCCGCATCT) and *PcSE*-R1 (CGGCGCTTTGAGGTCAACGA) were used for targeted knockout validation.

### Functional analysis of the PcSE extract

The effect of *PcSE* on the triterpene content of *P. cocos* was evaluated by UHPLC analysis. The *P. cocos* knockout strains of mycelium were collected and dried at 60 ℃ in the oven for 3 h to obtain a constant weight. The sample was ground into a fine powder using a mortar and pestle. Subsequently, 0.2 g of powder was added into 2 mL of anhydrous ethanol and let to stand for 1 h. The mixture was then extracted through ultrasonication (KM-500DB, 40 K Hz) for 2 h with clock shaking every 10 min. The crude extracts were stewed for 1 h and filtered through a 0.22 μm membrane filter for UHPLC-Q-TOF-MS analysis. A series of standard solutions of nine triterpene acids, namely HDTRA, PAB, DTUA, PAA, PAC, DPA, PAAM, AHDTRA and PA, were prepared as reference standards for determining the triterpene content of the mutant strains following findings from our previous study [12, 13].

### UHPLC-Q-TOF–MS analysis of PcSE

UHPLC-Q-TOF-MS analysis was performed by reversed-phase method using an Agilent 1290 liquid chromatography system (Agilent 6530, Agilent Technologies, Santa Clara, CA, USA) linked to an electrospray ionization-QTOF/MS detector (QExactive, Thermo, Waltham, MA, USA). UHPLC separations were performed using a C18 reversed-phase column (InertSustain C18 column, 5 μm, 4.6 × 250 mm, GL Sciences Inc. Tokyo, Japan). The column temperature was maintained at 40 °C, the flow rate was 1 mL min^−1^, and the detection wavelengths were set at 210 nm and 245 nm. The mobile phase comprised acetonitrile (A) and 0.1% formic acid in ultrapure water (B). The gradient elution settings were as follows: 0–5 min, 0–51% A; 5–30 min, 51–72% A; 30–37 min, 72–86% A; 37–47 min, 86–100% A; 47–60 min, 100% A. The extracted samples were quantified using a 20.0 μL injection volume. Target compounds were identified using UHPLC-MS/MS. Nitrogen was used as the drying and nebulizing flow gas at a flow rate of 10 L min^−1^. The drying temperature was 350 ℃ and the nebulizing gas pressure was 35 psi. High-purity helium was used as the collision gas with a collision energy of 30, 40, and 50 eV. The mass spectrometry (MS) scans ranged from m/z 100 to 600. The ESI interface was operated in the negative ion mode.

## Results

### PcSE gene sequence cloning and bioinformatics analysis

The genome of *Wolfiporia cocos* MD-104 SS10 has been released in 2012 (NCBI: ASM2944865v1) [7]. In the present work, we identified and cloned the *SE* of *P. cocos* and the PCR products were sequenced by TA cloning. The results showed that the cloned *PcSE* genome sequence was 1570 bp long with four exons and three introns. Analysis of the sequence revealed that the ORF of the *PcSE* gene is 1413 bp long, encoding a protein of 470 amino acid residues. Sequence analysis showed that the *PcSE* had a specific binding domain for *SE*. This finding implies that *PcSE* has squalene epoxidase function. Protein model prediction was conducted to obtain the 3D structure of the protein and molecular docking to identify potential ligands. The results showed that several amino acid sites are active and able to bind to the compound.

A homology model was built using AlphaFold and the quality of the model was evaluated (Additional file 1: Fig. S1). The results showed that 100% of the amino acid residues were in the rational region (including 94.7% in the optimal region, 5.3% in the acceptable region, and 0.0% in the general permissive region) and 0.0% in the disallowed region. These findings indicate that the model was accurate and reliable based on the evaluation principle that the reasonable region plus the allowed region should be greater than 90%. It was found through the ERRAT validation model that validation of the model showed a score of 96.214, indicating that it met the requirements of validation that state that the score should be greater than 80%. The evaluation scores showed that the constructed protein structure had high reliability and could be used as a template for subsequent studies.

**Fig. 1:**
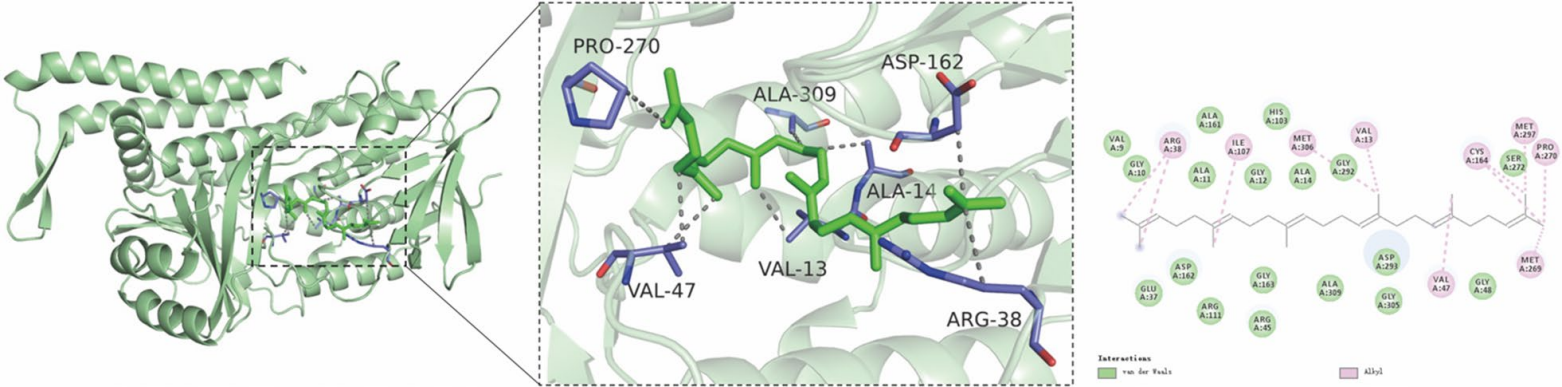
Protein model prediction and molecular docking poses of *Poria cocos* squalene epoxidase gene

The binding energy score of the docked ligand was − 7.4 kcal/moL, which indicated that the compound had a high binding affinity to the protein. PyMOL 2.3.0 was used for the analysis of the docking results. The results showed that the *PcSE* bound stably to the cavity of the protein and interact with the surrounding amino acids. Further analysis showed that the compound interacted with the protein mainly through hydrophobic interaction forces (Fig. 1). The hydrophobic groups in the compound formed hydrophobic interactions with amino acids VAL13, ALA14, ARG38, VAL47, ASP162, PRO270 and ALA309 in the protein, ensuring stable binding of the compound to the active site. This protein is implicated in the synthesis of 2–3 oxidized squalene in the *P. cocos* triterpene synthesis pathway.

### Plasmids construction

**Fig. 2:**
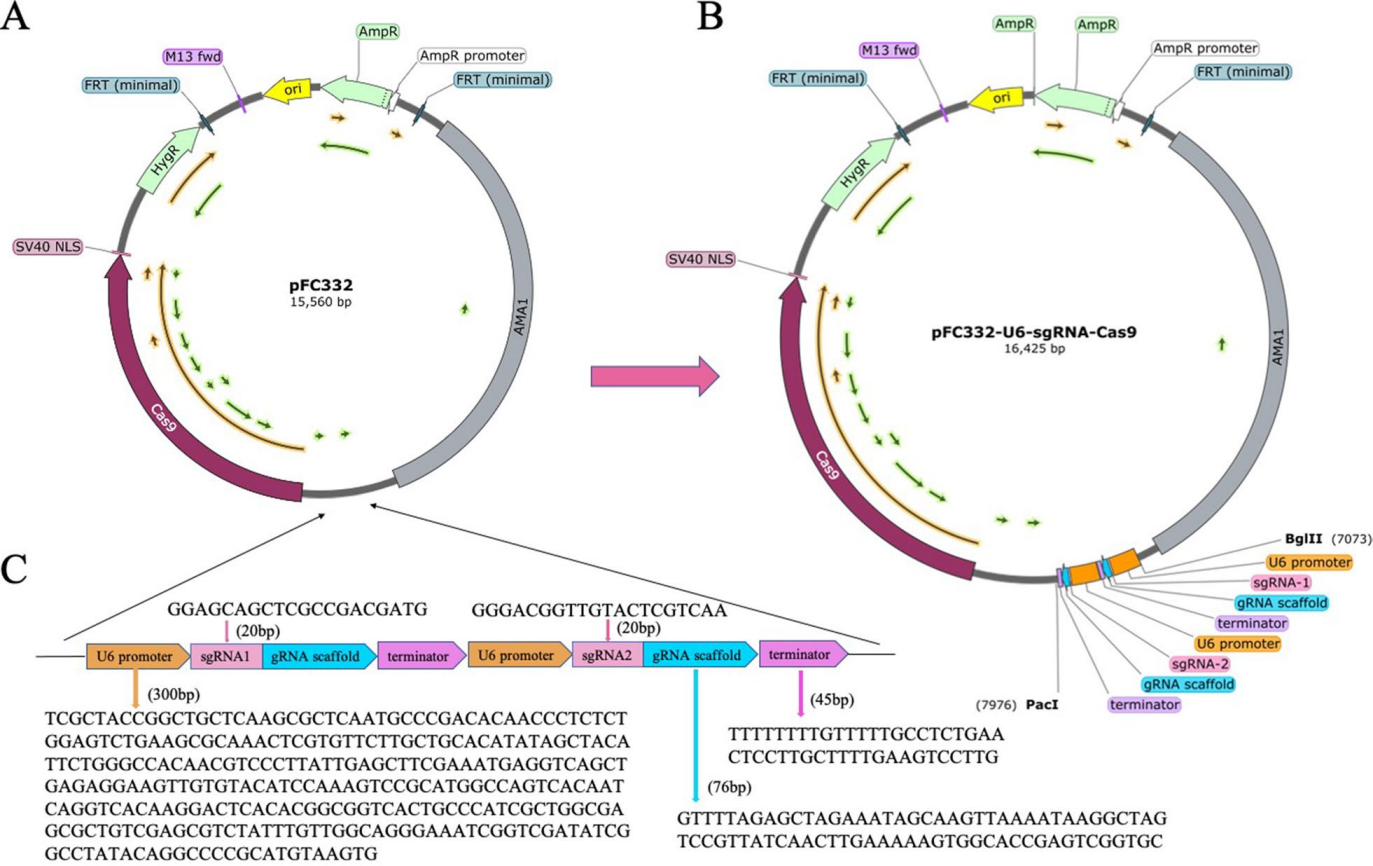
Construction of *Poria cocos* squalene epoxidase gene knockout plasmid vector. **A** pFC332 empty plasmid. **B** A complete pFC332-U6-sgRNA_*PcSE*_-Cas9 knockdown vector containing two sgRNA loci was successfully constructed. **C** The fragment and its amino acid sequence were inserted into the construct vector

We performed promoter optimization to improve the efficiency of gene editing. Three U6 snRNA messages were identified in the annotation file of *P. cocos* genome. The sequences were extracted using TBtools software and 1000 bp upstream of the first sequence and 1000 bp downstream of the second and third sequences were selected as potential promoter regions. Further, 500 bp downstream of the first sequence and 500 bp upstream of the second and third sequences were selected as potential terminator sequences. Promoter analysis for each of the three potential promoter sequences was performed using the gene promoter Miner (GPMiner) tool. A 300 bp sequence was then selected as the *P. cocos* endogenous U6 promoter. The terminator region was screened according to the homology and the continuous T rule at the 5' end and 4 more T bases were inserted at its downstream end as the termination signal. Ultimately, a 45 bp sequence was identified as the terminator region. Amino acids containing VAL-47 with a major effect on compound binding to the active site were selected as sgRNA1 and one sequence without amino acids with major effect was selected as sgRNA2 based on the molecular docking results to reduce the off-target rate and homologous recombination repair. The two specific sgRNA knockout sites were simultaneously inserted into the same knockout vector to ensure a successful knockdown of the other site in case one is knocked down and then repaired. A *P.cocos* gene editing knockout vector pFC332-U6-sgRNA_*PcSE*_-Cas9 (Fig. 2) containing two specific sgRNAs (20 bp), *P.cocos* endogenous U6 promoter (300 bp), terminator (45 bp) and gRNA scaffold (76 bp) was successfully constructed through DNA sequencing.

### Targeted deletion of the PcSE gene in P. cocos pFC332-U6-sgRNAPcSE-Cas9

To activate sgRNA elements in vivo, we optimized the endogenous U6 promoter of *P. cocos* to improve the efficiency of the Cas9 expression vector for knockdown of specific genes. Knockdown of the *SE* gene in *P.cocos* was achieved using a double sgRNA-directed CRISPR/Cas9 system following the editing method of *Ganoderma lucidum* using double sgRNA for the *ura3* gene [16, 17]. Analysis showed that knockdown of the target genes occurred in the sgRNA1 locus containing VAL-47 residue found at the active site. The results showed no deletion or insertion of the target gene in the sgRNA2 knockout sites that did not contain VAL-47 amino acid. This can be attributed to the direct effect of the knockout sites with active amino acids on the function of *PcSE*, making it easier to knockdown the gene. These results indicate that optimization of *P. cocos* endogenous initiation region and use of two specific sgRNA loci achieves targeted editing of squalene cyclooxygenase gene in *P. cocos*.

**Fig. 3:**
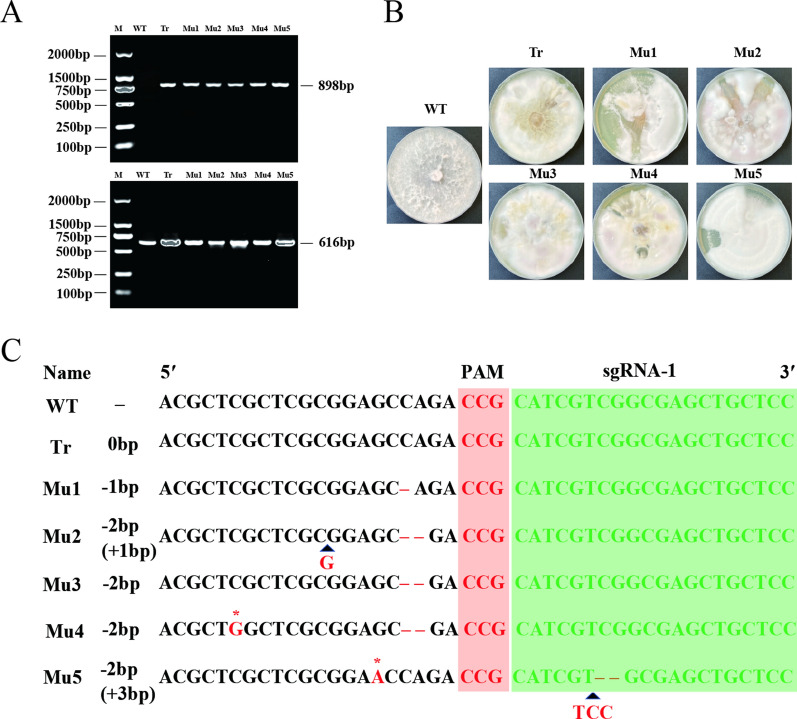
Editing of *Poria cocos* squalene epoxidase gene by a dual-sgRNA-directed CRISPR/Cas9 system through PEG-mediated protoplast transformation. WT denotes the wild-type strain, Tr represents transformed un-knocked strains, Mu1-Mu5 are mutants. **A** Verification of the sgRNA1 deletion strain by PCR. M, DL 2 000 DNA marker; 898 bp represents transfer-in plasmid using primers for validation and 616 bp for validation using knockout primers. **B** Screening and re-selection of ergosterol-resistant mutants after sgRNA1 targeting *SE* were inserted into transformant pFC332-U6-2-sgRNA_*PcSE*_-Cas9. **C** DNA sequences of the target fragment of the sgRNA1 knockout strains. The red and green boxes indicate the PAM and guide sequence, respectively; + indicates inserted fragments,—indicates deleted fragments; Mu1, 1 bp deletion; Mu2, 1 bp insertion and 2 bp deletion; Mu3, 2 bp deletion; Mu4, 1 bp substitution and 2 bp deletion; Mu5, 4 bp deletion, 1 bp substitution and 3 bp insertion, respectively

To perform the targeted deletion of *SE* in *P. cocos* using the CRISPR/Cas9 system, we transformed the vector plasmid with double sgRNA into *P. cocos* protoplasts and transformants were screened on resistant plates containing hygromycin and ergosterol. The selected transformants were then cultured in non-selective medium for five generations of succession. Agarose gel electrophoresis of genomic DNA extracted from the transformed and wild strains was performed to validate the PCR products (Fig. 3A). Use of specific primers for vector plasmids showed that the wild strains did not have transfer plasmids, whereas the transformed strains had knockout plasmids. Clear and bright bands were observed when using the sequence verification primers for *PcSE*, indicating that the PEG-mediated protoplast method was effective in performing transformation of *PcSE*. The results showed that the transformed strain and the mutant strain had dense mycelium with some differences after 5 generations of succession culture phenotypes (Fig. 3B). For example, the mycelium of the transformed strain was denser and the aerial mycelium was relatively less compared with the wild-type strain. Moreover, the distribution of mycelium in the transformed strain was not as uniform as that of the wild-type strain. The folds were thicker at the region where the mycelium of the transformed strain made contact with the culture medium.

**Fig. 4:**
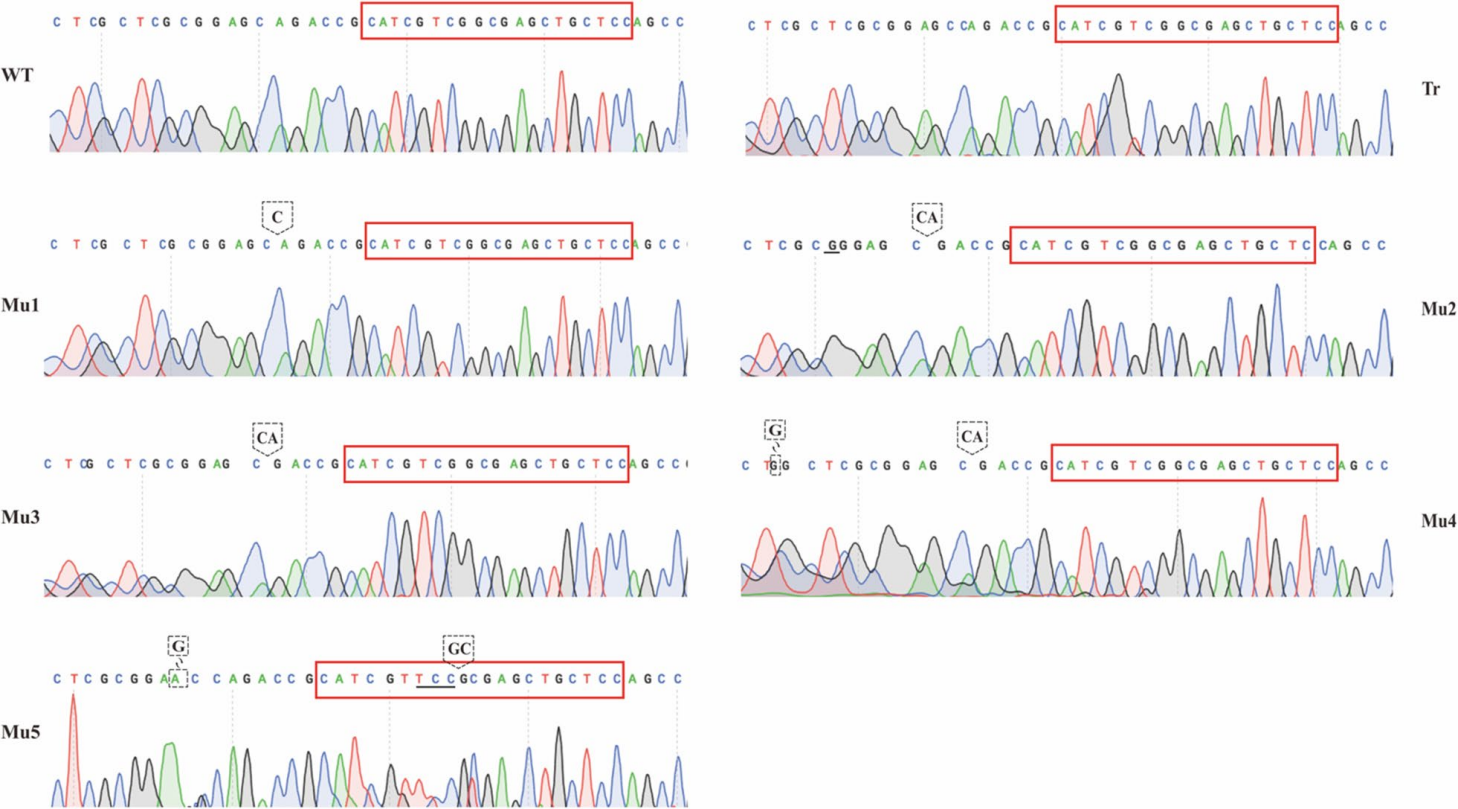
Sequencing peak map of *Poria cocos* squalene cyclooxygenase gene. WT denotes the wild-type strain, Tr represents the transformed un-knocked strain, and Mu1-Mu5 are the mutants. Red boxes indicate sgRNA1 bootstrap sequences; the underlines indicate inserted bases; arrow dashed boxes indicate missing bases; superimposed dashed boxes indicate substitution bases

DNA sequencing was performed based on the PCR results to verify whether editing of *PcSE* occurred (Figs. 3C, 4). The results showed that no fragment deletion of *PcSE* occurred in transformed strain 1, indicating that the CRISPR/Cas9-based system did not edit the transformed strain 1 (Tr) and no mutation occurred in *P.cocos* strain. A deletion of *PcSE* was detected at the sgRNA1 locus in transformed strains 2, 3, 4, 5, and 6, and mutant strains were obtained (Mu1, Mu2, Mu3, Mu4 and Mu5). A deletion of 1 bp was observed in Mu1; an insertion of 1 bp and a deletion of 2 bp in Mu2; a deletion of 2 bp in Mu3; a substitution of 1 bp and a deletion of 2 bp in Mu4; and a deletion of 4 bp, a substitution of 1 bp, and an insertion of 3 bp were observed in Mu5. These findings indicate that these strains can be used for targeted knockdown of *PcSE* at the sgRNA1 locus using a CRISPR/Cas9-mediated gene editing system. These results indicate that the CRISPR/Cas9 system is an effective tool for gene deletion in *P. cocos*.

### Changes in triterpenoid content in P. cocos mutant strains

**Fig. 5:**
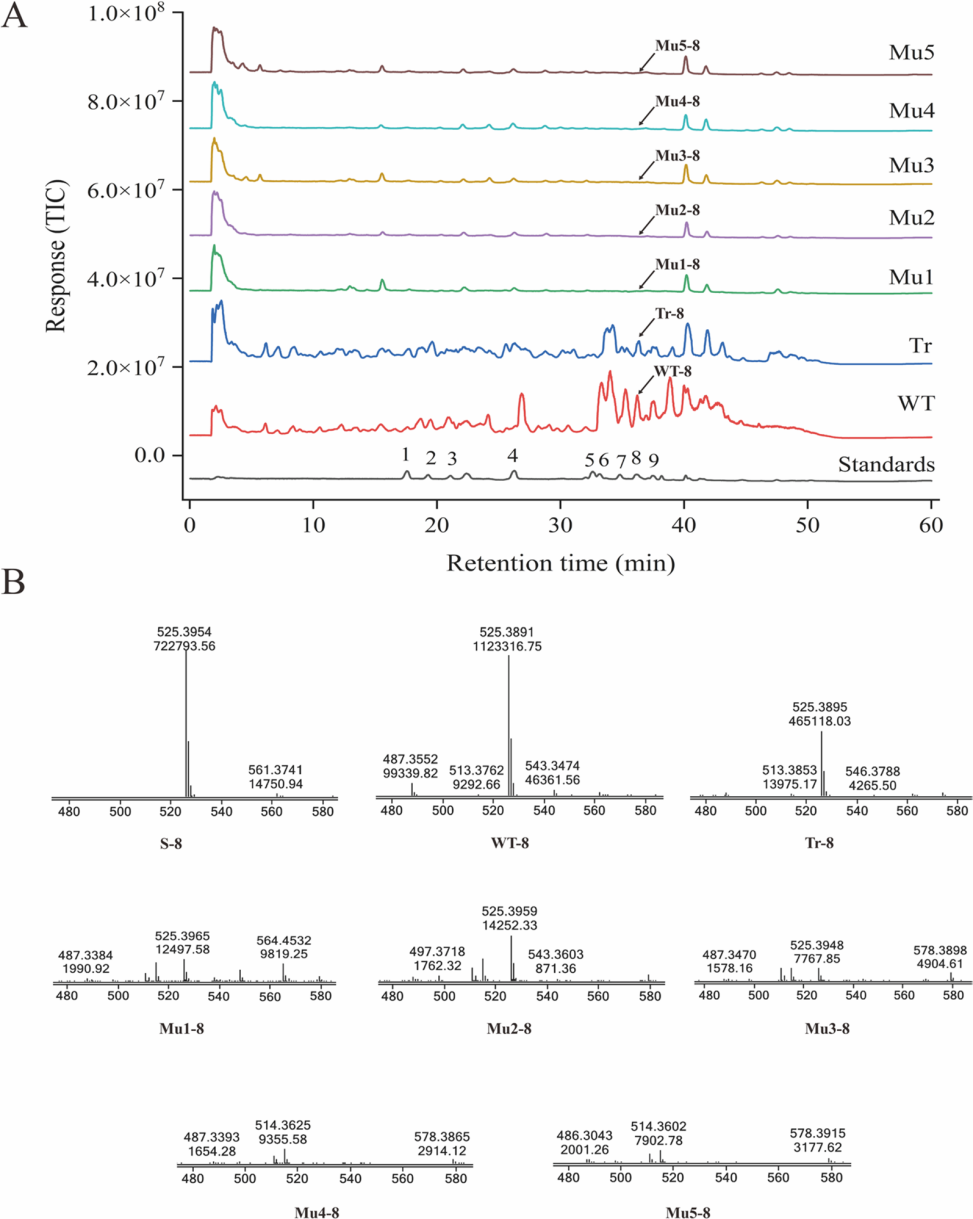
Analysis of *Poria cocos* mutant strains by ultra-high-performance liquid chromatography-quadruple time of flight-dual mass spectrometry (UHPLC-QTOF-MS/MS) analysis. **A** Total ion chromatography (TIC) of *P. cocos* mutant strains and triterpene acid standards. WT denotes the wild-type strain, Tr represents the transformed un-knocked strains, Mu1-Mu5 indicate the mutants. 1, 16α-hydroxydehydrotrametenolic acid (HDTRA); 2, poricoic acid B (PAB); 3, dehydrotumulosic acid (DTUA); 4, polyporenic acid C (PAC); 5, poricoic acid AM (PAAM); 6, 3-O-acetyl-16α-hydroxydehydrotrametenolic acid (AHDTRA), 7, 3-O-acetyl-16α-hydroxytrametenolic acid (AHTRA); 8, dehydropachymic acid (DPA); 9, pachymic acid (PA). **B** The MS/ MS spectrum of dehydropachymic acid (DPA) from HPLC-QTOF-MS/MS

**Fig. 6:**
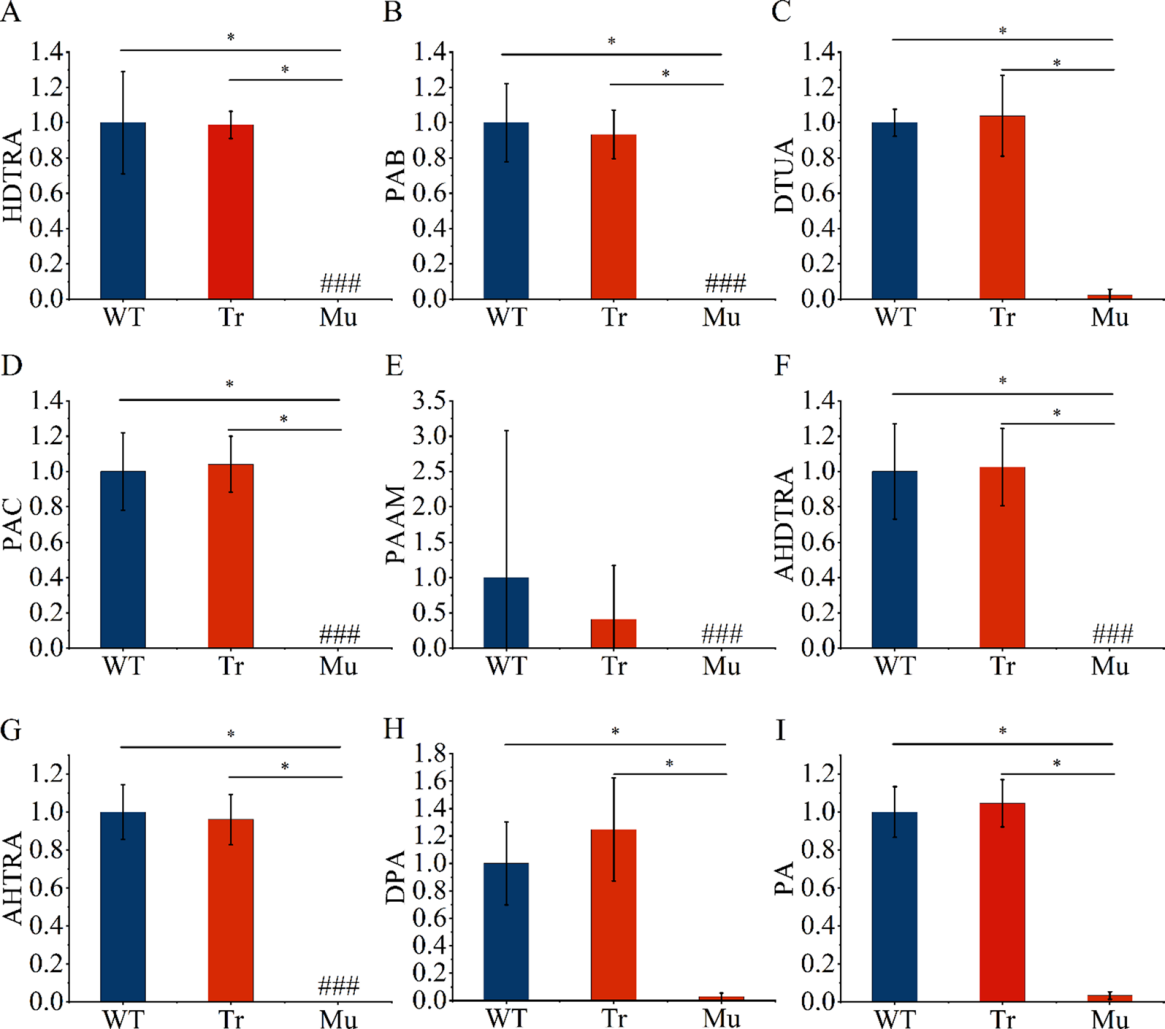
Relative abundance of nine triterpene components from *Poria cocos*. WT represent the wild-type strains, Tr represents transformed un-knocked strains, and Mu denote the mutants. *, *p*-value < 0.05. ###, no detection

The triterpene composition of coconut mutant strains was evaluated using UHPLC-QTOF-MS/MS under the negative ion mode using nine standards, namely HDTRA, PAB, DTUA, PAC, PAAM, AHDTRA, AHTRA, DPA and PA. These substances are downstream products of wool sterols in the *P. cocos* triterpene synthesis pathway, and the knockout of *SE* would theoretically have an impact on the content of these components. The LC–MS total ion chromatograms (TIC) are shown in Fig. 5A. Nine triterpene components were detected in the strain transfected with the plasmid without gene knockout compared with the wild strain. Six triterpene components identified in the strains that had not undergone knockout of the gene were not detected in the knockout mutant strains. Only trace amounts of the other three triterpene components, namely DTUA, DPA, and PA, were detected in the knockout mutant strains. Mass spectrometric (MS) analysis of DPA (Fig. 5B) showed the presence of DPA in the mutant strains Mu 1, Mu2 and Mu3 but absent in mutant strains Mu4 and Mu5. Although DTUA, DPA and PA were detected in some strains, the relative levels of these compounds in the mutant strains were extremely low compared with the wild strains and the non-knockout strains (Fig. 6). These showed that the triterpene contents of the wild strain and the transformed non-knockout strain were significantly higher than those of the mutant strain. The results showed that deletion of *PcSE* inhibited the synthesis of *P. cocos* triterpenes, demonstrating that *PcSE* has a squalene epoxidase function.

## Discussion

*SE* is an essential component for the synthesis of 2–3-epoxysqualene by squalene epoxidase, and plays a crucial role in triterpene synthesis pathway [14]. From previous studies, we can learn that *SE* gene research has been carried out in species such as *Panax ginseng* [9], *Tripterygium wilfordi*i [16, 17, 38], *Taraxacum koksaghyz* [23]and *Betula platyphylla*(M. [36, 37]. Many other functional genes in the *P. cocos* triterpene synthesis pathway have been studied successively [24, 25, 40], but no *PcSE*-related reports are available. Research on the *PcSE* gene is crucial for the application of secondary metabolites in *P. cocos* and for regulating the synthesis of triterpenes in *P. cocos* at the molecular level. The establishment of an efficient gene editing system is an effective means to solve this problem.

The use of single or multiple specific sgRNAs to inactivate entire genes during gene function validation can verify their function, for example in *Cordyceps militaris* [18] and *Ganoderma lucidum* [16, 17]. However, the above methods do not clarify the structural features of the function, especially the key active site regions of the function. In this study, the conformational relationship between small molecule ligands and protein targets was predicted using the molecular docking technique of virtual screening [4] to obtain amino acid active sites for *PcSE* gene sequences. The predicted active amino acid sites were subsequently combined with conventional design approaches to obtain specific sgRNA sequences with amino acid active sites. The results showed that base insertions, substitutions and deletions were implemented at the sgRNA1 site containing the amino acid active site. In contrast, no significant editing was seen in sgRNA2 without amino acid active site, indicating that molecular docking successfully predicted the active functional domain of the *PcSE* gene. It also showed that the base sequence of the selected functional active site was designed as sgRAN to facilitate targeted editing of the gene.

We successfully knocked out the *PcSE* gene using sgRNA with active amino acid site. This is the first time that the CRISPR/Cas9 system combined with molecular docking technology has been used to edit *PcSE*. Not only was the gene functionally verified, but the functional active site was also identified. UHPLC-QTOF-MS/MS analysis of *P. cocos* mutants revealed that knockdown of the *PcSE* gene inhibited the synthesis of *P. cocos* triterpene acids. We developed a molecular docking-based sgRNA suitable for target gene knockdown in medicinal edible fungi.

## Conclusion

In this study, we successfully cloned and edited the *PcSE* gene, and constructed an efficient gene editing system in *P. cocos*. A 3D model of *PcSE* sequence was constructed to improve the efficiency of the CRISPR system and the active amino acid sites were predicted using molecular docking techniques. A sgRNA containing the active amino acids was established and inserted into the Cas9 expression vector together with the endogenous promoter of *P. cocos.* PEG-mediated protoplasts were used to transform *P. cocos* and the expression vector containing double sgRNA. Finally, we successfully obtained the mutant strain with the deletion of *PcSE*. The function of squalene epoxidase in the *P. cocos* mutant strain was verified using in vivo experiments.

### Supplementary Information

**Additional file 1: Figure S1.** Protein model evaluation of *Poria* *cocos* squalene epoxidase. **A** Protein models; **B** and **C.** Protein models score.

## Data Availability

Data will be made available on request.

## Abbreviations

CRISPR: Clustered regularly interspaced short palindromic repeats
*PcSE*: Squalene epoxidase of *Poria cocos*
UHPLC-QTOF-MS/MS: Ultra-high-performance liquid chromatography-quadruplex time-of-flight-double mass spectrometry
TIC: Total ion chromatograms
sgRNA: Single chimeric guide RNA
ZFN: Zinc finger nuclease technology
TALEN: Transcriptional activator like effector nuclease technology
WT: Wild type
Tr: Transformed un-knocked strains
Mu1-Mu5: Mutants 1–5
LB: Luria–Bertani
HDTRA: 16α-Hydroxydehydrotrametenolic acid
PAB: Poricoic acid B
DTUA: Dehydrotumulosic acid
PAC: Polyporenic acid C
DPA: Dehydropachymic acid
PAAM: Poricoic acid AM
AHDTRA: 3-O-acetyl-16α-hydroxydehydrotrametenolic acid
AHTRA: 3-O-acetyl-16α-hydroxytrametenolic acid
PA: Pachymic acid
MS: Mass spectrometry
