# Supplementary material for: Identification of squalene epoxidase in triterpenes biosynthesis in Poria cocos by molecular docking and CRISPR-Cas9 gene editing
Source: Microb Cell Fact. 2024 Jan 25;23:34. doi: 10.1186/s12934-024-02306-3 (PMC10809676; doi:10.1186/s12934-024-02306-3)
Supplement: Supplementary file 1 — Additional file 1: Figure S1. Protein model evaluation of Poria cocos squalene epoxidase. A Protein models; B and C. Protein models score. [file 12934_2024_2306_MOESM1_ESM.docx]

**Supporting file**

Additiona data to this article can be found online.


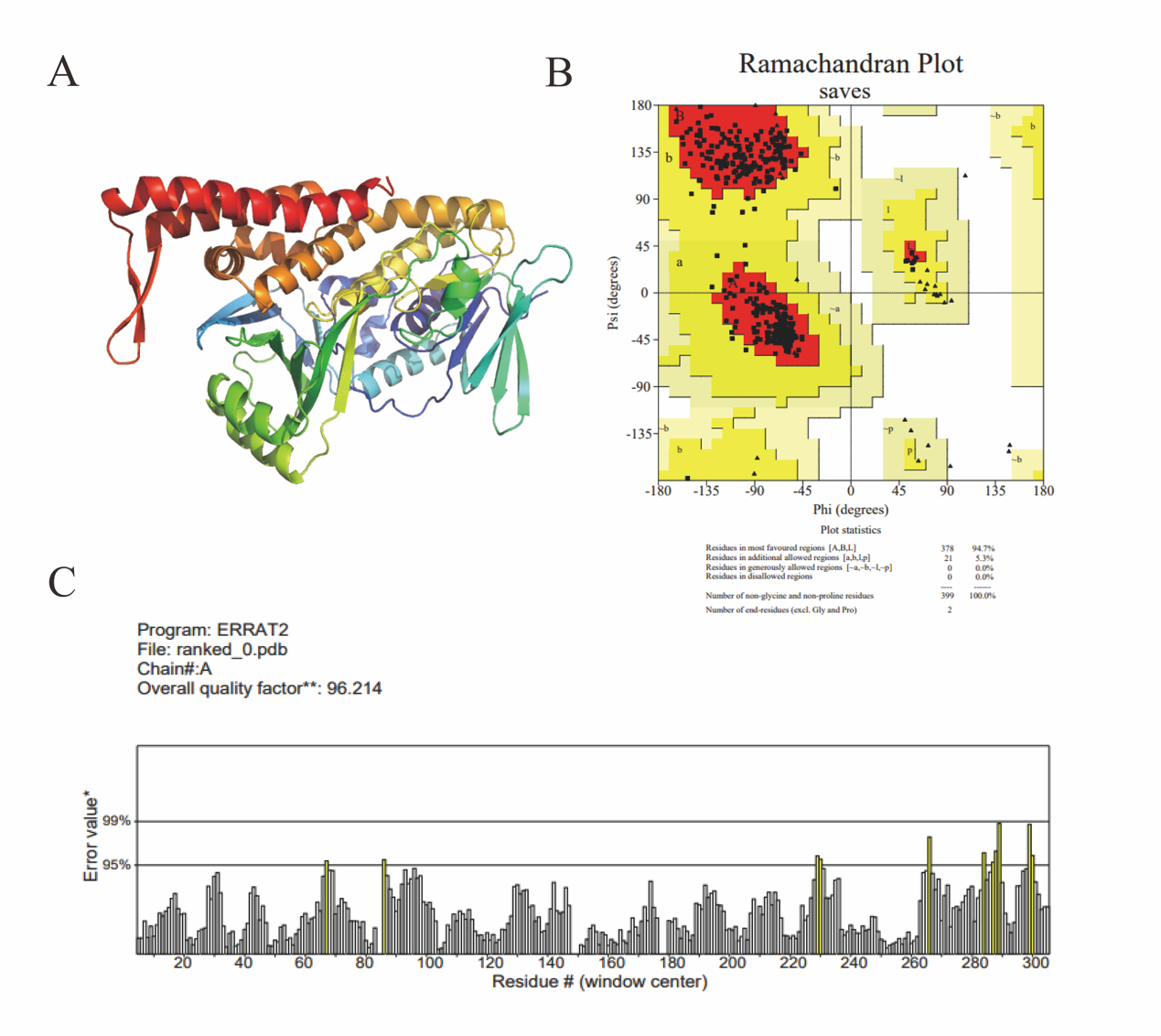


Fig. S1. Protein model evaluation of *Poria cocos* squalene cyclooxygenase gene. A. Protein models; B and C. Protein models score.
